# Identification of a Siglec-F+ granulocyte-macrophage progenitor

**DOI:** 10.1002/JLB.1MA1217-475R

**Published:** 2018-04-12

**Authors:** Jessica E. Bolden, Erin C. Lucas, Geyu Zhou, Jeremy A. O’Sullivan, Carolyn A. de Graaf, Mark D. McKenzie, Ladina Di Rago, Tracey M. Baldwin, Jake Shortt, Warren S. Alexander, Bruce S. Bochner, Matthew E. Ritchie, Douglas J. Hilton, Kirsten A. Fairfax

**Affiliations:** 1 The Walter and Eliza Hall Institute of Medical Research, Parkville, Victoria, Australia; 2 Department of Medical Biology, The University of Melbourne, Parkville, Victoria, Australia; 3 Division of Allergy and Immunology, Department of Medicine, Feinberg School of Medicine, Northwestern University, Chicago, Illinois, USA; 4 School of Clinical Sciences at Monash Health, Monash University, Clayton, Victoria, Australia

**Keywords:** eosinophil, granulocyte, hematopoiesis, Myb, neutrophil

## Abstract

In recent years multi-parameter flow cytometry has enabled identification of cells at major stages in myeloid development; from pluripotent hematopoietic stem cells, through populations with increasingly limited developmental potential (common myeloid progenitors and granulocyte-macrophage progenitors), to terminally differentiated mature cells. Myeloid progenitors are heterogeneous, and the surface markers that define transition states from progenitors to mature cells are poorly characterized. Siglec-F is a surface glycoprotein frequently used in combination with IL-5 receptor alpha (IL5R*α*) for the identification of murine eosinophils. Here, we describe a CD11b^+^ Siglec-F+ IL5R*α*− myeloid population in the bone marrow of C57BL/6 mice. The CD11b^+^ Siglec-F+ IL5R*α*− cells are retained in eosinophil deficient PHIL mice, and are not expanded upon overexpression of IL-5, indicating that they are upstream or independent of the eosinophil lineage. We show these cells to have GMP-like developmental potential in vitro and in vivo, and to be transcriptionally distinct from the classically described GMP population. The CD11b+ Siglec-F+ IL5R*α*− population expands in the bone marrow of *Myb* mutant mice, which is potentially due to negative transcriptional regulation of Siglec-F by Myb. Lastly, we show that the role of Siglec-F may be, at least in part, to regulate GMP viability.

## 1 | INTRODUCTION

Myeloid differentiation begins from multipotential long-term and short-term hematopoeitic stem cells, progresses in a step-wise fashion to yield progenitor cells with increasingly limited developmental potential, and ends with the generation of terminally differentiated cells with specialized function. The use of multicolor flow cytometry has led to the description of many distinct progenitors that have multi-, oligo-, and unipotent outputs. For example, granulocyte-macrophage progenitors (GMPs) are capable of eventually giving rise to mature neutrophilic and eosinophilic granulocytes and monocyte/macrophages, but have lost the potential to make lymphoid, megakaryocytic, and erythroid cells.^1^ More restricted progenitors include the common monocyte progenitor (cMoP),^2^ the eosinophil-lineage committed progenitor (EoP),^3^ and the basophil/mast cell progenitor (B/MCP).^4,5^ The isolation of each of these progenitors has begun to establish the developmental steps taken in the generation of myeloid cells, and revealed some of the key transcription factors that control these processes, nevertheless transitional intermediates and the surface markers that identify them during myelopoiesis are largely undefined.

The transcription factor c-Myb is a critical regulator of hematopoiesis. Indeed, *Myb* knockout mice die at E15.5 due to severe hematopoietic defects,^6^ and mice harboring hypomorphic *Myb* alleles (*Myb*^Plt4/Plt4^ mice) have numerous hematopoietic alterations, including supraphysiological platelet production and defects in lymphoid specification.^7,8^ Myb regulates many key genes in myeloid cells (neutrophil elastase, *Spi1* (Pu.1), *Cebpb*, and *Runx1*)^9–12^ and *Myb*^Plt4/Plt4^ mice have increased granulocyte/macrophage colony forming capacity,^7^ thus Myb regulates myeloid development.

Siglecs (sialic acid binding, immunoglobulin-like lectins) are a family of cell-surface glycoproteins expressed primarily by innate immune cells.^13,14^ Siglec-F and its human functional paralog Siglec-8, are key surface markers used for the identification of eosinophils.^15,16^ Both Siglec-F and Siglec-8 can be engaged with sialylated glycans (natural and synthetic) or antibodies to induce eosinophil death,^17–20^ although the extent to which Siglec-F-induced apoptosis controls tissue eosinophilia is dependent upon the experimental model used.^21^ The natural tissue ligands for Siglec-F include glycan derivatives from Muc5b, a mucin that is constitutively expressed on tracheal epithelial cells.^18^ Both *SiglecF* and *Muc5b* knockout mice have enhanced allergic eosinophilic inflammation following allergen challenge,^18,22^ suggesting that Muc5b glycans and Siglec-F constitute a negative feedback pathway that helps resolve eosinophilic inflammation. Siglec-F is also expressed on alveolar and peritoneal macrophages,^23,24^ mast cells and dendritic cells in the intestine,^25,26^ and intestinal epithelial (tuft and M) cells,^27,28^ although the function of Siglec-F in these contexts is unclear.

Here, we describe a myeloid progenitor population that expresses Siglec-F, but unlike EoPs, is IL-5-receptor alpha (IL5R*α*) negative. We show these progenitors to have GMP-like developmental potential in vitro and in vivo, but to be transcriptionally distinct from canonical GMPs. Furthermore, we demonstrate a potential role for Siglec-F in the regulation of GMP viability.

## 2 | MATERIALS AND METHODS (BRIEF)

Full details are provided as supplementary methods. Summaries are provided below:

### 2.1 | Mouse strains

All procedures involving mice were approved by the WEHI Animal Ethics Committee or the Institutional Animal Care and Use Committee of Northwestern University. UBC-GFP mice^29^ were obtained from the Jackson Laboratory. *Myb^Plt4^* mice are described in Ref. 7. PHIL mice (C57BL/6) were provided by Drs. James and Nancy Lee.^30^ IL-5 transgenic (IL-5Tg) mice^31^ were backcrossed on to a C57BL/6 background.

### 2.2 | Flow cytometry

Bone marrow (BM), spleens, blood, and peritoneal cavity lavage cells were collected from 8–12 week old mice. Red blood cells were removed by lysis with an ammonium chloride-based buffer. Cells were stained with cocktails of antibodies recognizing CD11b, IL5R*α*, Siglec-F, B220, CD3, Ly6C, Ly6G, ST2, Lin, cKit, Scal, Fc*γ*RII/III, CD34, and CD45. Cells were resuspended in PBS/2% FCS, 2 mM EDTA, 1 *μ*g/ml propidium iodide (Sigma) to enable identification and exclusion of dead cells. Cells were analyzed on a BD LSR Fortessa X-20 flow cytometer (BD Biosciences).

Cell populations were defined using the following surface markers: Eosinophils (CD11b+ Siglec-F+ IL5R*α*^Int^SSC^Hi^), CMP (Lin-cKit + Sca1-CD34 + Fc*γ*RII/III^Lo^), GMP (Lin-cKit + Sca1-CD34 + Fc*γ*RII/III+), and EoP (Lin-cKit + Sca1-CD34 + Fc*γ*RII/III + IL5R*α*+).

Flow cytometric analyses were performed with FlowJo V10 software (FlowJo). Statistical tests and graphs were generated with Prism (GraphPad Software).

### 2.3 | Cytocentrifuge preparations

Sorted cells were cytocentrifuged onto glass slides, air dried, fixed with 100% methanol, and stained with May Grünwald’s stain (Merck) and 5% Giemsa in pH 6.8 buffered water (Merck) according to manufacturer’s instructions.

### 2.4 | Colony forming assays

Cells were sorted from the BM of C57BL/6 mice. Colony assays were performed as described in Ref. 32 and scored by viewing on a Nikon Optiphot-2 light microscope.

### 2.5 | In vivo developmental potential assays

BM was flushed from 13 week old UBC-GFP mice in PBS/2% FCS, overlaid onto 60% Percoll, and centrifuged at 400 × *g* for 25 min. Cells at the interface were collected and stained for IL5R*α*, Siglec-F, CD11b, and CD34 expression. CD11b+ Siglec-F+ IL5R*α*− cells were sorted, washed with ice-cold PBS and resuspended in ice-cold PBS.

Recipient Ly5.1 mice were irradiated (550 rad) 24 h prior to transplantation. Sorted cells (25 *μ*L) were injected under the capsule of the sinus of the spleen. A mock recipient mouse was injected with 25 *μ*l PBS. Spleens were collected from recipient mice 1 and 3 d after surgery and prepared for flow cytometry.

### 2.6 | In vivo Siglec-F ligation

Mice (8–12 week old) were injected with 20 *μ*g anti-Siglec-F 9C7 antibody (a gift from Dr. James Paulson, The Scripps Research Institute) or rat IgG2b isotype control antibody (LTF-2, Tonbo Biosciences), i.p, every second day, on 4 occasions. BM was collected 24 h after the last injection and prepared for flow cytometry.

### 2.7 | RNA-sequencing

Populations were sorted from the BM of 6–10 week old C57BL/6 and 6 week old *Myb^Plt4/Plt4^* mice. Total RNA was isolated using the RNAeasy Micro Kit (Qiagen). Overall, 130–200 ng total RNA per sample was submitted to the Australian Genome Research Facility for high throughput mRNA-sequencing. Libraries (mRNA) were synthesized using Illumina’s TruSeq Stranded mRNA protocol, and 100 bp reads generated with an Illumina HiSeq 2500 (Illumina). Two to five independent RNA samples per cell type were sequenced. Bioinformatic analyses are detailed in supplementary methods.

## 3 | RESULTS AND DISCUSSION

### 3.1 | A Siglec-F+ IL5R*α*− population is present in the BM of wildtype mice

**FIGURE 1:**
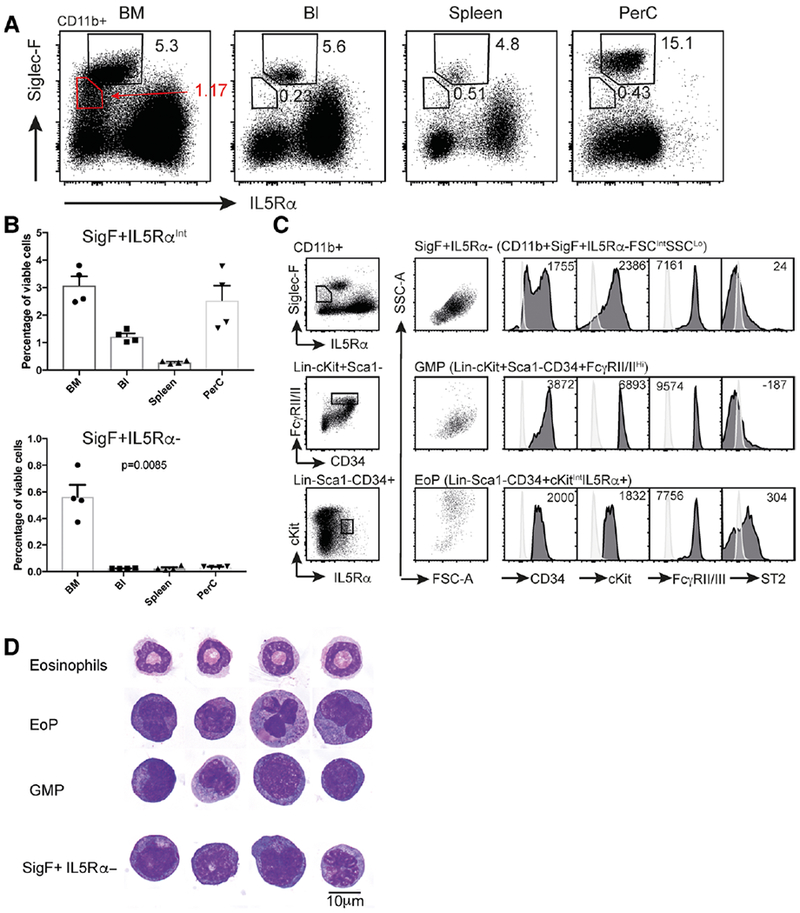
Identification and characterization of Siglec-F+ IL5R*α*− cells. (A) Flow cytometry dot plots showing Siglec-F and IL5R*α* expression in CD11b+ viable cells in C57BL/6 bone marrow (BM), blood (Bl), spleen, and peritoneal cavity lavage fluid (PerC). Eosinophils (Siglec-F+ IL5R*α*^Int^) and Siglec-F+ IL5R*α*− populations are gated. The percentage of cells falling within each gate are shown. (B) Quantification of Siglec-F+ IL5R*α*^Int^ (eosinophil) and CD11b+ Siglec-F+ IL5R*α*− cells (as a percentage of viable cells) in the 4 tissues shown in (A). Note, contaminating eosinophils in the CD11b+ Siglec-F+ IL5R*α*− gate have been excluded from quantification on the basis of high side scatter. Data is presented as mean + sem (*n* = 4 mice), with individual mouse data points shown. *p*-values determined by one way ANOVA (C). Light scatter and surface marker expression in the CD11b+ Siglec-F+ IL5R*α*− population, GMPs and EoPs. Cells in the surface marker histograms for the Siglec-F+ IL5R*α*− population have been pregated to exclude any contaminating eosinophils on the basis of high side scatter. Light colored histogram represents the fluorescence of unstained BM cells. Numbers indicate the average geometric mean fluorescence for each surface marker from 4 independent mice (D) Representative images of sorted and cytocentrifuged populations following May Grunwald Giemsa staining. Ten micrometer scale bar shown

Siglec-F is a signature surface protein found on eosinophils that is used in many laboratories to identify these cells. We have identified a population of cells that is Siglec-F+ but is IL5R*α*− and CD11b+ (Fig. 1A). These cells make up 0.56 ± 0.09% (mean ± SEM) of viable cells in the BM, with very few cells falling in this gate in the peripheral blood (BI), spleen (Spl), or peritoneal cavity (PerC) (0.02 ± 0.001%, 0.02 ± 0.007%, and 0.04 ± 0.001%, respectively) (Fig. 1A and B). As eosinophils are typically IL5R*α*+ and are known to expand in response to IL-5, we sought to characterize this Siglec-F+ IL5R*α*− population further. The Siglec-F+ IL5R*α*− population has distinct FSC properties, being larger than lymphocytes, eosinophils, or neutrophils (Supplementary Fig. 1A and B), and side scatter (SSC) properties that are similar to neutrophils, that is having lower SSC than eosinophils and higher SSC than lymphocytes (Supplementary Fig. 1A and C). Overall, 78.5 ± 1.6% of the cells express CD34, and the cells are cKit^Int^, ST2−, Fc*γ*RII/III+ (Fig. 1C). Morphologically, these cells have nuclei that in cytocentrifuge preparations appear ellipsoid or monocytoid, with occasional cells exhibiting nuclear segmentation, indicating they are in the myeloid lineage, which is consistent with the CD11b staining (Fig. 1D). Upon May Grünwald Giemsa staining, they have a moderate amount of basophilic (purple/blue) cytoplasm and morphologically resemble GMPs.

### 3.2 | The Siglec-F+ IL5R*α*− population is present in PHIL mice and is not expanded in IL-5Tg mice

**FIGURE 2:**
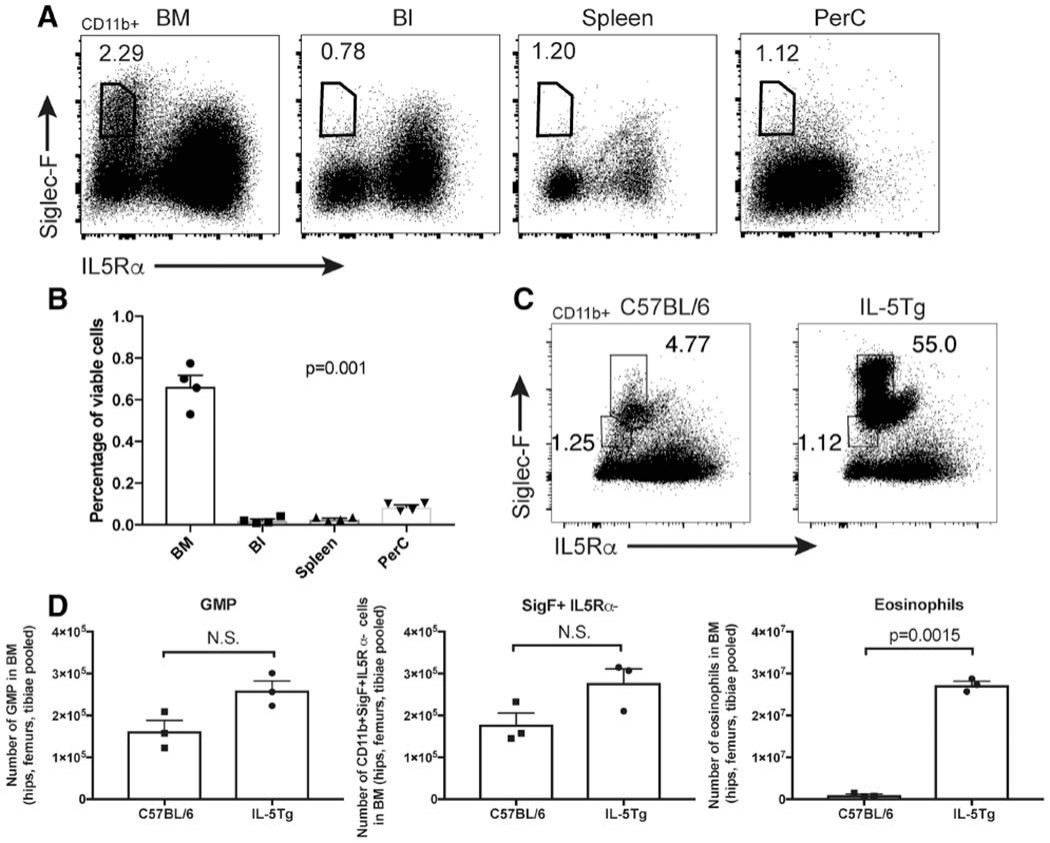
The Siglec-F+ IL5R*α*− population is present in PHIL mice and is not expanded in IL-5Tg mice. (A) Flow cytometry dot plots showing Siglec-F and IL5R*α* expression in CD11b+ cells in bone marrow (BM), blood (BI), spleen and peritoneal cavity lavage fluid (PerC) from eosinophil-deficient PHIL mice. The Siglec-F+ IL5R*α*− cell population is gated, and percentage of gated (CD11b+) cells shown. (B) Quantification of CD11b+ Siglec-F+ IL5R*α*− cells (as a percentage of viable white blood cells) in the tissues shown in (A). *p* = 0.001 as determined by one-way ANOVA. (C) Representative flow cytometry dot plots of Siglec-F and IL5R*α* expression in CD11b+ cells isolated from the BM of C57BL/6 and IL-5Tg mice. The percentage of cells falling into the Siglec-F+ IL5R*α*^Int^ (Eosinophil) and Siglec-F+ IL5R*α*− gates (as a percentage of CD11b+ cells) are shown. (D) Quantification of the number of GMP, Siglec-F+ IL5R*α*− cells, and eosinophils in BM of C57BL/6 and IL-5Tg mice. Data are presented as mean + sem (*N* = 3 or 4). Individual mouse data points are shown. *p*-values were determined by an unpaired two-tailed Student’s *t*-test with Welch’s correction and have been corrected for multiple comparisons using the method of Bonferroni. N.S, not significant

Siglec-F+ IL5R*α*− cells do not have scatter or morphological characteristics of eosinophils, however, they do express the eosinophil marker Siglec-F. We therefore formally tested whether Siglec-F+ IL5R*α*− cells fall within the eosinophil lineage. PHIL mice are devoid of eosinophils and their precursors (EoPs) due to transgenic expression of Diphtheria Toxin A from the Eosinophil Peroxidase promoter, which becomes transcriptionally active in EoPs.^30^ PHIL mice retained the Siglec-F+ IL5R*α*− cells, and, similar to wildtype mice, there were approximately 10-fold more Siglec-F+ IL5R*α*− cells in the BM than in any of the peripheral organs examined (Fig. 2A and B). Siglec-F+ IL5R*α*− cells are therefore not EoPs, nor are they derived from an Epx-expressing progenitor.

IL-5 is a potent stimulator of eosinophil development, and IL-5Tg mice have marked eosinophilia and expansion of EoPs^31^ (Fig. 2C and D). Consistent with their lack of IL5R*α* expression, Siglec-F+ IL5R*α*− cells were not significantly expanded in IL-5Tg mice (Fig. 2C and D). Together these data suggest that it is unlikely that Siglec-F+ IL5R*α*− cells lie downstream of an IL-5-responsive precursor; rather they are likely to lie upstream of an EoP or are of a lineage unrelated to eosinophils.

### 3.3 | The Siglec-F+ IL5R*α*− population has predominantly neutrophilic developmental potential in vitro and in vivo

Given the morphological similarity of the Siglec-F+ IL5R*α*− cells to GMPs, and their surface expression of cKit, we assessed whether sorted CD11b+ Siglec-F+ IL5R*α*− cells had proliferative potential.

**FIGURE 3:**
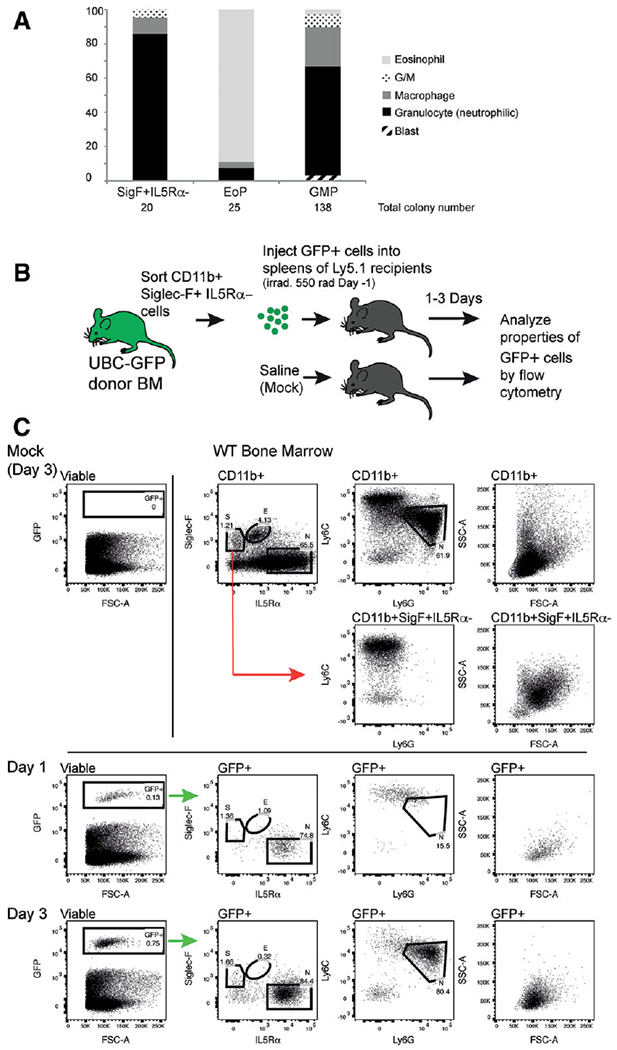
CD11b+ Siglec-F+ IL5R*α*− cells predominantly develop into non-eosinophil granulocytes in vitro and in vivo. (A) In vitro colony forming assay showing the percentage of each colony type formed when CD11b+ Siglec-F+ IL5R*α*−, EoP and GMP populations are plated in soft agar with stem cell factor + IL3 + EPO. The total numbers of colonies formed by each cell type are indicated. G/M indicates mixed granulocyte/macrophage colonies. (B) Schematic representation of in vivo developmental potential assay (C) In vivo developmental potential assay. **Top right** panels show typical staining characteristics of CD11b+ cells in bone marrow. The positions of eosinophils (E), neutrophils (N) and the injected CD11b + Siglec-F+ IL5R*α*− (S) population are shown on the Siglec-F/IL5R*α* dot plot. Ly6C/Ly6G and FSC/SSC profiles of total CD11b+ cells are shown. The position of neutrophils (N) in terms of Ly6G and Ly6C expression is indicated. The Ly6C/Ly6G and FSC/SSC characteristics of gated CD11b+ Siglec-F+ IL5R*α*− cells are also shown. **Top left** panel shows lack of GFP+ cells in a mock (PBS)-injected spleen, 3 days after surgery. **Third row,** the identification of GFP+ cells from the spleen 1 day post injection, and the surface marker expression and scatter characteristics of GFP+ cells. **Bottom row,** the identification of GFP+ cells from the spleen 3 days post injection, and the surface marker expression and scatter characteristics of GFP+ cells. The predicted positions of the starting population (S), eosinophils (E), and neutrophils (N), based on side-by-side staining of WT BM (top row), are shown

We tested the colony-forming potential of Siglec-F+ IL5R*α*− cells in semi-solid agar cultures with a cocktail of EPO, stem cell factor, and IL-3, which facilitates the development of erythroid, granulocyte, eosinophil, macrophage, megakaryocyte, and blast colonies. Siglec-F+ IL5R*α*− and GMPs both made granulocytic, macrophage, and granulocyte/macrophage colonies (Fig. 3A), with the GMPs having a higher colony output than the Siglec-F+ IL5R*α*− population. GMPs also generated a small number of blast and eosinophil colonies when plated at this density. The Siglec-F+ IL5R*α*− population also had the potential to make eosinophil colonies when plated at a higher cell density (data not shown). Sorted EoPs (95% purity) made almost exclusively eosinophil colonies.

To examine their in vivo developmental potential, we sorted CD11b+ Siglec-F+ IL5R*α*− cells from the BM of GFP+ mice and transplanted them into the spleens of irradiated Ly5.1 (GFP negative) recipients. The immunophenotype and morphology of GFP+ cells in the spleen were analyzed by flow cytometry 1 and 3 days post-surgery (Fig. 3B). No GFP+ cells were detected in mice that were injected with PBS alone (mock). GFP+ cells recovered from the spleens of transplanted mice had upregulated surface IL5R*α* by day 1, and by day 3 80% resembled neutrophils (IL5R*α*+Ly6G+Ly6C^Int^SSC^Int^FSC^Int^, Fig. 3C). Together, these data demonstrate that CD11b+ Siglec-F+ IL5R*α*− cells have GMP-like developmental potential in vitro, and favor the production of granulocytes/neutrophils in vivo. The loss of surface Siglec-F on transplanted cells suggests that Siglec-F expression in GMPs is transient and subsequently suppressed upon differentiation.

### 3.4 | The Siglec-F+ IL5R*α*− population is prominent in *Myb* mutant mice

**FIGURE 4:**
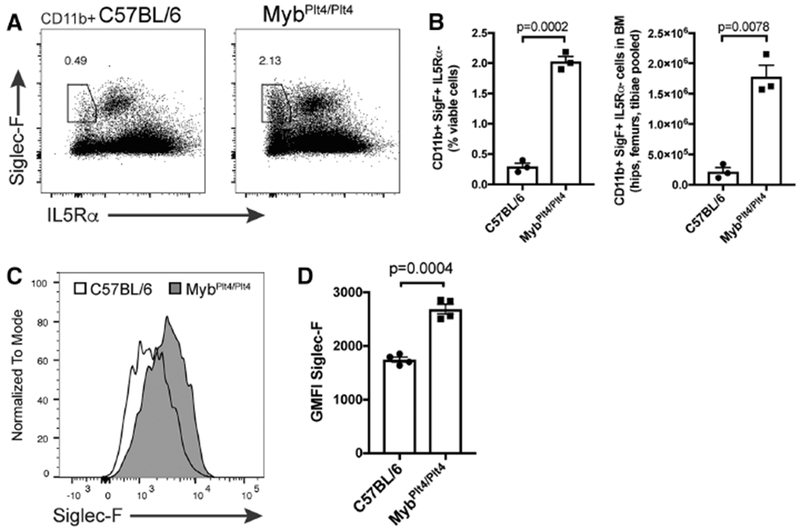
CD11b+ Siglec-F+ IL5R*α*− cells increase in the bone marrow of *Myb* mutant mice. (A) Siglec-F and IL5R*α* expression in CD11b+ cells from the bone marrow of C57BL/6 and *Myb*^Plt4/Plt4^ mutant mice, as determined by flow cytometry. (B) Quantification of the percentage (of viable cells) and numbers of CD11b+ Siglec-F+ IL5R*α*− cells in C57BL/6 and *Myb*^Plt4/Plt4^ BM. (C) Siglec-F expression in GMPs from representative C57BL/6 and *Myb^Plt4/Plt4^* mice (D) Quantification of geometric mean fluorescence intensity (GMFI) of Siglec-F expression in GMPs from C57BL/6 and *Myb^Plt4/Plt4^* mice. *p*-values were determined by an unpaired, two-tailed Student’s *t*-test with Welch’s correction

*Myb* knockout mice die during early embryonic development due to severe hematopoietic defects.^6^ Hypomorphic *Myb* mutant mice (*Myb^Plt4/Plt4^*) generated in our laboratory have increased granulocyte/macrophage colony forming capacity.^7^ As shown in Fig. 4A and B, *Myb^Plt4/Plt4^* mice also exhibited a striking increase in the percentage and numbers of CD11b + Siglec-F+ IL5R*α*− cells in the BM. Published ChIP analyses have shown the promoter of Siglec5 (encoding Siglec-F) to be occupied by Myb.^12^ We examined the surface expression of Siglec-F on GMPs from C57BL/6 and *Myb^Plt4/Plt4^* mice (Fig. 4C and D). GMPs have broad Siglec-F expression, which is elevated in Myb mutant cells. These data suggest that Myb negatively regulates Siglec-F expression, and provides a possible mechanism for the prominence of Siglec-F+ GMPs in *Myb^Plt4/Plt4^* mice.

### 3.5 | Siglec-F ligation regulates Siglec-F+ GMP viability and Siglec-F internalization

**FIGURE 5:**
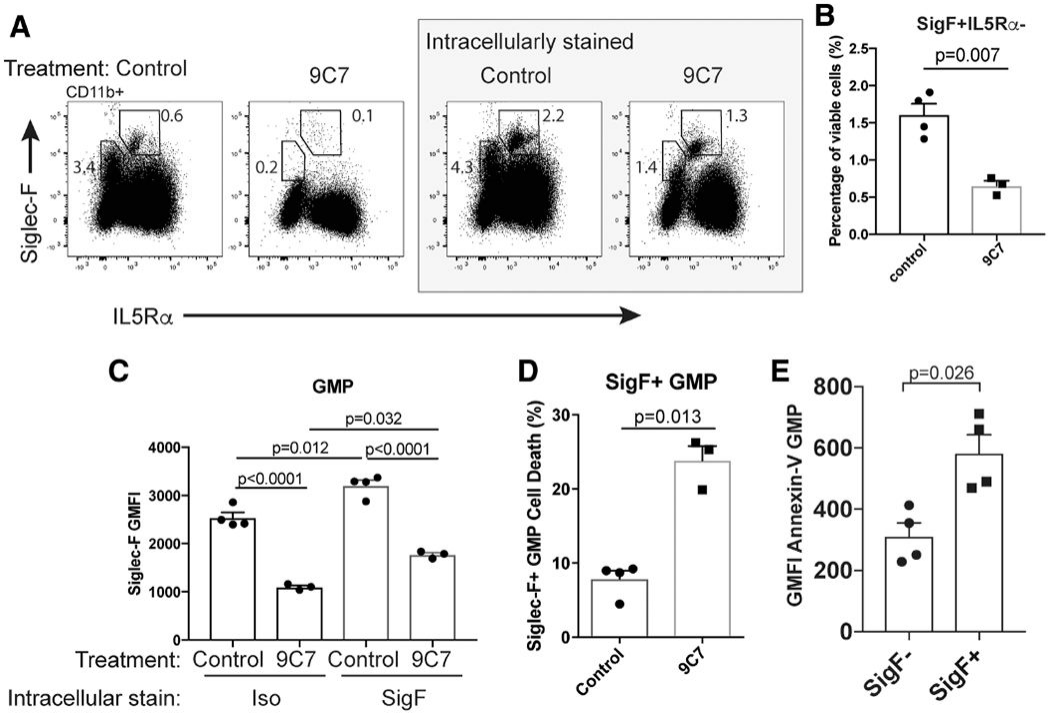
In vivo Siglec-F ligation with antibody affects Siglec-F+ GMP viability. C57BL/6 mice were injected i.p. with anti-siglec-F (9C7) or isotype (control) antibodies. (A) Representative Siglec-F and IL5R*α* expression in 9C7 and control antibody treated mice (pre-gated CD11b+), following intracellular isotype staining (i.e., surface Siglec-F expression, left panels) and intracellular Siglec-F staining (right panels). (B) Quantification of bone marrow CD11b+ Siglec-F+ IL5R*α*− cells following 9C7 or isotype treatment and intracellular Siglec-F staining. (C) Siglec-F fluorescence intensity in GMPs from 9C7 and control treated mice following surface (Iso) and intracellular Siglec-F staining. (D) Induction of Siglec-F+ GMP cell death following 9C7 treatment. (E) Quantification of Annexin-V binding in untreated Siglec-F+ and Siglec-F− GMPs. *p*-values in (B), (D), and (E) were determined by an unpaired, two-tailed Student’s *t*-test with Welch’s correction, and further correction for multiple testing. *p*-values in (**C**) were determined by a one-way ANOVA with multiple testing correction using Sidak’s method

As engagement of Siglec-F with antibodies can induce modest degrees of eosinophil death in vitro, and a decrease in the number of eosinophils in vivo,^33^ we explored whether CD11b+ Siglec-F+ IL5R*α*− cells are affected by Siglec-F ligation. In vivo administration of the anti-Siglec-F antibody 9C7 caused loss of surface Siglec-F expression from CD11b+ cells in the BM, as measured by flow cytometry (Fig. 5A and B). The eosinophil population was restored by Siglec-F intracellular staining (demonstrating Siglec-F internalization), however, CD11b+ Siglec-F+ IL5R*α*− cells were only partially recovered with this technique (Fig. 5A and B). The additional loss of CD11b+ Siglec-F+ IL5R*α*− cells following anti-Siglec-F treatment could be explained by three mechanisms: (i) death of Siglec-F+ cells, (ii) shedding or downregulation of Siglec-F, and (iii) interference between 9C7 and the E50-2440 clone used for detection. Thus, while anti-Siglec-F treatment did not lead to a statistically significant decrease in eosinophils (as determined by intracellular staining with anti-Siglec-F), it caused a reduction in CD11b+ Siglec-F+ IL5R*α*− cells (*p* = 0.007).

In addition, we examined the effect of 9C7 on classically gated GMPs. 9C7 treatment reduced surface Siglec-F expression and increased internalization of Siglec-F in GMPs (Fig. 5C). We found a small but statistically significant increase in dead Siglec-F+ GMPs following 9C7 treatment (Fig. 5D). Consistent with this finding, Siglec-F+ GMPs have higher levels of Annexin-V binding (in the absence of ligation), an event typically associated with early apoptosis (Fig. 5E). Together, these data suggest that Siglec-F ligation can at least partially regulate the viability of Siglec-F+ GMPs.

### 3.6 | The Siglec-F+ IL5R*α*− population is transcriptionally distinct from both classical GMPs and EoPs

**FIGURE 6:**
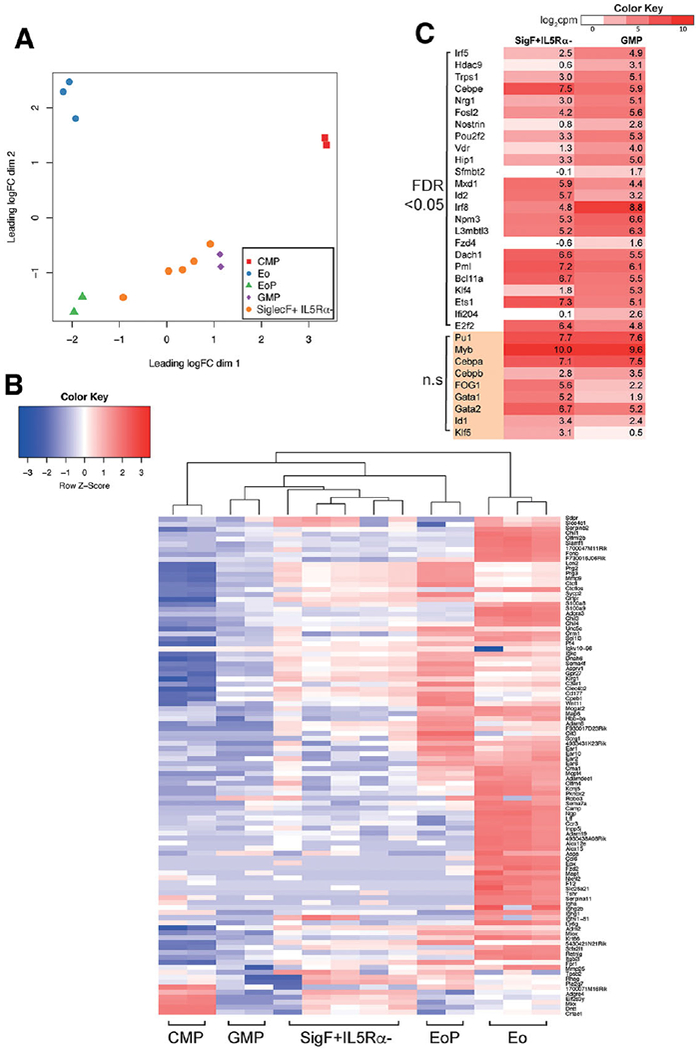
Siglec-F+ IL5R*α*− cells are transcriptionally distinct from classical GMPs. (A) Multidimensional scaling plot of the RNA-seq profiles from common myeloid progenitor (CMP), granulocyte-macrophage progenitor (GMP), eosinophil-lineage restricted progenitor (EoP), eosinophil (Eo), and Siglec-F+ IL5R*α*− populations based on the 500 most variable genes between each pair of samples. (B) Heatmap and clustering of the top 100 most variable genes across the cell types shown in (A). The heatmap is colored according to row scaled gene expression (log_2_ cpm). (C) Heatmap of transcription factor expression in Siglec-F+ IL5R*α*− cells and GMPs. Factors are grouped according to significance (FDR < 0.05, and not-significant n.s FDR > 0.05). The heatmap is color-coded by the average log_2_ counts per million

Our data suggest that Siglec-F+ IL5R*α*− cells are a distinct subpopulation with myeloid potential. Using RNA-sequencing, we profiled gene expression in sorted populations of myeloid progenitors (CMP, GMP, EoP), Eos, and Siglec-F+ IL5R*α*− cells. When we compared the transcriptional profile of each of these subsets using a multidimensional scaling (MDS) plot, the Siglec-F+ IL5R*α*− cells were distinct from each of the classical populations and exhibited a profile intermediate between GMPs and EoPs (Fig. 6A). Consistent with these analyses, clustering of the 100 most variable genes (across all samples shown in the MDS plot) also positioned the Siglec-F+ IL5R*α*− population most closely to GMPs (Fig. 6B) with the detectable expression of a subset of early EoP/eosinophil genes, including *Prg2* and *Prg3*, but lacking expression of *Epx* and eosinophil associated RNases.

As the Siglec-F+ IL5R*α*− cells had GMP-like developmental potential, we examined the gene expression differences between this population and canonical GMPs. We found 120 genes to be significantly upregulated in the Siglec-F+ IL5R*α*− population (FDR < 0.05); with 230 genes that were downregulated in the Siglec-F+ IL5R*α*− population, relative to GMPs (350 genes in total, Supplementary Table 1). This contrasted with the comparison between the Siglec-F+ IL5R*α*− population and EoPs, where > 14-fold more genes were differentially expressed (DE); 3005 genes upregulated and 2143 downregulated (Supplementary Table 2). Therefore, transcriptionally, the Siglec-F+ IL5R*α*− cells more closely resemble GMPs than EoPs.

Given the similarities in morphology, and transcriptional profiling between GMPs and the Siglec-F+ IL5R*α*− population, we conducted gene ontology (GO) analyses on the 350 DE genes to better understand differences between the two populations.^34,35^ The most statistically significant GO terms associated with DE genes included ‘immune_system_process’ (FDR *q*-value (*q*) = 1.21 × 10^−54^, 101 genes are marked in Supplementary Table 1) and ‘immune_response’ (*q* = 4.32 × 10^−36^). Highly significant was the term ‘regulation_of_cytokine_production’ (*q* = 4.3 × 10^−23^), with *Csf1R, IL6R, C3* and *Tlr5*, and *Tlr8* among the 38 DE genes captured by this category.

Having demonstrated Siglec-F+ IL5R*α*− cells are expanded in the *Myb^Plt4/Plt4^* mice, we performed RNAseq on this population from *Myb^Plt4/Plt4^* mice and controls and identified 813 DE genes between Siglec-F+ IL5R*α*− populations from *Myb^Plt4/Plt4^* and C57BL/6 WT mice (Supplementary Table 3). Of the 416 genes more highly expressed in *Myb^Plt4/Plt4^* cells, 90 were involved with ‘immune_system_process’ (*q* = 2.22 × 10^−37^) and 54 with ‘biological_adhesion’ (*q* = 2.42 × 10^−24^). Twelve genes associated with integrin signaling, including seven integrins, were upregulated in *Myb^Plt4/Plt4^* cells, suggesting potentially altered binding, adhesion or migratory capabilities. We examined the DE lists for known Myb target genes (based on Myb ChIP data produced in the Snyder lab at Stanford: ENCODE data available online as ENCSR000ETR, and data published by the Gonda lab with a truncated Myb^12^) finding that 43% of genes upregulated in *Myb^Plt4/Plt4^* cells were known Myb ChIP targets, compared to 49% of downregulated genes, although the mechanisms underlying regulation of the population by Myb requires further study.

We analyzed the transcription factors^36^ that were differentially expressed between the Siglec-F+ IL5R*α*− population and GMPs. Eight transcription factors were upregulated in the Siglec-F+ IL5R*α*− population (*Cebpe, Mxd1, Id2, Dach1, Pml, Bcl11a, E2f2*, and *Ets1*) and 16 downregulated in the Siglec-F+ IL5R*α*− population compared to GMPs (including *Irf5, Irf8, Nrg1, Klf4*, and *Ifi204*). Expression of these factors, together with a suite of transcriptional regulators known to be important for GMP specification are shown in Fig. 6C.

We particularly noted the differential expression of the transcription factor Irf8 (higher in GMPs than the Siglec-F+ IL5R*α*− population) (Fig. 6C). Olsson et al. recently described a large-scale single cell RNA-sequencing study performed on sorted GMPs and described Irf8 and Gfi1 as negatively correlated, concluding that Irf8^hi^ cells were monocytic precursors, and Irf8- (Gfi1^hi^) were specified granulocyte and bipotential precursors.^37^ Although this level of specification was not observed in our experiments, we did observe that there was a bias towards the formation of granulocytes by CD11b+ Siglec-F+ IL5R*α*− cells in our in vitro colony assays compared to GMPs. The downregulation of *Irf8* in the Siglec-F+ IL5R*α*− population was concomitant with downregulation of *Klf4, Irf5*, and *Csf1r* as would be predicted by the Olsson et al. study (*Klf4* and *Irf5* shown in Fig. 6C). Drissen et al.^23^ also recently performed single cell RNA-sequencing experiments reporting heterogeneity within myeloid progenitors. Specifically, Drissen et al. described a Gata1-positive GMP that was biased towards eosinophil and mast cell specification, and a Gata1-negative GMP biased towards neutrophil and monocyte specification. Our Siglec-F+ IL5R*α*− population has a higher mean expression of Gata1 than GMPs but the expression difference is not statistically significant and the cells are not specified towards eosinophil generation (Fig. 6C).

In sum, we have described a GMP-like population that expresses Siglec-F, is transcriptionally distinct from GMPs and has predominantly neutrophilic granulocyte potential in cell production assays. Given the increase in Annexin-V staining within the Siglec-F+ GMP gate we propose that Siglec-F may function, at least in part, to regulate viability of this GMP subset.

## Supplementary Material

Supplement 1

Supplement 2

Supplement 3

Supplement 4

## Abbreviations:

BI: peripheral blood
BM: bone marrow
ChIP: chromatin immunoprecipitation
cMoP: common monocyte progenitor
CMP: common myeloid progenitor
cpm: counts per million
DE: differentially expressed
Eo: eosinophil
EoP: eosinophil-lineage committed progenitor
EPO: erythropoietin
FDR: false discovery rate
FSC: forward scatter
GMP: granulocyte-macrophage progenitor
GSEA: gene set enrichment analysis
hi: High
IL5R*α*: IL-5 receptor alpha (CD125)
IL-5Tg: IL-5 transgenic
Int: intermediate
lin: lineage
lo: low
MDS: multidimensional scaling plot
PerC: peritoneal cavity lavage fluid
PHIL: eosinophil-deficient strain
Spl: spleen
SSC: side scatter
