# Supplementary material for: Identification of a Siglec-F+ granulocyte-macrophage progenitor
Source: J Leukoc Biol. Author manuscript; Available in PMC 2019 Jan 5. (PMC6320667; doi:10.1002/JLB.1MA1217-475R)
Supplement: Supplement 1 [file NIHMS1003894-supplement-Supplement_1.pdf]

Supplementary Extended Methods

Mouse strains:

All procedures involving mice were approved by The Walter and Eliza Hall Institute of Medical Research Animal Ethics Committee or the Institutional Animal Care and Use Committee of Northwestern University.

C57BL/6 and Ly5.1 mice were sourced from the WEHI Clive and Vera Ramaciotti Laboratories. UbiquitinC promoter-green fluorescent protein (UBC-GFP) mice (C57BL/6-Tg(UBC-GFP)30Scha/J) [1], were obtained from the Jackson Laboratory. Myb^{Plt4} mice were generated in-house in an ENU mutagenesis screen and are described by Carpinelli *et al*. [2]. PHIL mice (backcrossed onto a C57BL/6 background) were kindly provided by Drs James and Nancy Lee [3]. IL-5 transgenic (IL5-Tg) mice were described by [4], and have been backcrossed onto a C57BL/6 background for at least 9 generations.

Flow cytometry and cell sorting:

Bone marrow (flushed from femurs, tibiae and hips, triturated through 23G needle), spleens (passed through 40μm cell strainer), peripheral blood (from the retro orbital sinus) and cells from the peritoneal cavity (obtained by lavage) were collected from 8-12 week old mice. For flow cytometry, red blood cells were removed by lysis with an ammonium chloride based buffer (156 mM NH_4Cl, 11.9 mM $NaHCO_3$, 0.097 mM EDTA). Cells, at a concentration of $2x10^7$/mL, were stained on ice with cocktails of antibodies recognising the following cell surface proteins on mature cells (CD11b, IL5Rα, Siglec-F, B220, CD3, Ly6C, Ly6G) and progenitors [Lin, cKit, Sca1, FcγRII/III (CD16/32), CD34, IL5Rα, Siglec-F, ST2]. Prior to flow cytometry, cells were resuspended in Phosphate-buffered saline (PBS) supplemented with 2% FCS, 2

mM EDTA, 1 μg/mL propidium iodide (Sigma) to enable identification and exclusion of dead cells. Stained cells were analyzed on a BD LSR Fortessa X-20 flow cytometer (BD Biosciences) or sorted on a BD FACS Aria III (BD Biosciences).

For cell sorting of CMP, GMP, EoP and Eos for RNAseq, red cells and other mature blood cells were removed by positive selection as follows: total bone marrow was incubated with a cocktail of antibodies against mature cell markers (including Ter119) and selected with Biomag goat anti-rat IgG magnetic beads (Qiagen). Flow-through cells (negative cells) were subsequently stained with fluorescently-conjugated antibodies against surface markers and sorted as described below. Prior to sorting of the Siglec-F+IL5Rα- population, Siglec-F+ cells were enriched through staining with a PE-conjugated anti-Siglec-F antibody, and positive selection with anti-PE microbeads (Miltenyi Biotec). Due to the fluorophore combinations used in this sort, we were unable to include an antibody against IL5Rα. The Siglec-F+IL5Rα- fraction was sorted using additional gates to exclude known IL5Rα+ populations on the basis of other markers, such as excluding Eos and EoPs on the basis of scatter. A terminal FSC^{Int} SSC^{Lo} gate was also applied to positively sort Siglec-F+IL5Rα- cells.

Known cell populations were defined using the following surface markers: Eosinophils (CD11b+, Siglec-F+, $IL5R\alpha^{Int}$, SSC^{High}), CMP (Lin-, cKit+, Sca1-, CD34+, $CD16/32^{lo}$), GMP (Lin-, cKit+, Sca1-, CD34+, CD16/32+), EoP (Lin-, cKit+, Sca1-, CD34+, CD16/32+, IL5Rα+). In the peritoneum, CD11b very high cells (very large, autofluorescent cells, presumably macrophages) had been excluded from downstream Siglec-F v IL5Rα flow cytometry dotplots through application of a CD11b+ gate (that excludes CD11b+ very high cells). $CD11b^{V.Hi}$ cells were not observed in the other organs analyzed.

Flow cytometric analyses were performed with FlowJo V10 software (FlowJo). Subsequent statistical tests (ANOVA, corrections for multiple comparisons using Sidak's method and unpaired Student's t tests with Welch's correction) and graphs were generated with Prism (GraphPad Software). Student's t test p-values were adjusted for multiple testing according the method of Bonferroni using the p.adjust function in R.

Annexin-V staining:

Cells were prepared and stained for flow cytometry as described above. After the antibody washes, cells were washed once with 1x Annexin-V binding buffer (10mM Hepes pH 7.4, 140mM NaCl, 2.5mM $CaCl_2$) and incubated in 1x Annexin-V binding buffer containing 1/100 Annexin-V-FITC (WEHI) and 1 μg/mL propidium iodide (Sigma) for 10 minutes at room temperature. Stained cells were analyzed by flow cytometry on an LSR Fortessa X-20 flow cytometer as described above.

In vivo anti-Siglec-F antibody injection:

Eight-to-twelve-week-old C57BL/6 mice were injected intraperitoneally every second day with 20 μg anti-Siglec-F (clone 9C7, a gift from Dr. James Paulson, The Scripps Research Institute, La Jolla, CA, USA) or rat IgG2b isotype control antibody (clone LTF-2, Tonbo Biosciences) a total of 4 times. Tissues were harvested 24 hours after the final administration. Bone marrow, blood and spleens were processed and stained for flow cytometry. Surface and intracellular anti-Siglec-F staining was performed with clone E50-2440 or IgG2a isotype control, and cell viability was assessed using Fixable Viability Dye eFluor 506 (Thermo Fisher Scientific). Following surface staining, cells were fixed and permeabilized with BD Cytofix/Cytoperm (BD

Biosciences). Cells were then washed and stained for intracellular Siglec-F in BD Perm/Wash buffer. Cells were analyzed on a BD LSR II flow cytometer (BD Biosciences).

Antibodies:

Cells were stained with the following rat anti-mouse monoclonal antibodies prior to flow cytometry: CD11b (M1/70, WEHI and BD Pharmingen), Siglec-F (E50-2440, BD Pharmingen and BD Horizon), IL5Rα (T21, BD Pharmingen), Ly6C (HK1.4, eBioscience), Ly6G (1A8, BD Pharmingen), B220 (RA3-6B2, BD Pharmingen and BD Horizon), CD3 (KT3-1-1, WEHI), cKit (2B8, Biolegend), Sca1 (D7, BD Pharmingen), CD16/32 (2.4G2, BD Horizon), CD34 (RAM34, BD Pharmingen), ST2 (RMST2-2, Thermo Fisher Scientific), CD45 (30-F11, Biolegend). Lineage stains comprised the following antibodies: CD3 (KT3-1-1, WEHI), CD4 (GK1.5, WEHI), CD8 (53-6-6, WEHI), B220 (RA3-6B2, WEHI), CD19 (1D3, WEHI), Gr1 (RB68C5, WEHI) and Ter119 (Ly-76, WEHI).

Cytocentrifuge preparations and May Grünwald Giemsa stains:

Sorted cells were cytocentrifuged onto glass slides using a Shandon Cytospin 3 cytocentrifuge (Thermo Fisher Scientific) for 5 min at 500 rpm. Slides were air dried, fixed with 100% methanol for 10 min and stained with May Grünwald's stain (Merck) for 5 min. Slides were immediately transferred into 5% Giemsa solution (in pH 6.8 buffered water, Merck) for 20 min, washed twice for 30 s in pH 6.8 buffered water, washed for 1 min in dH_2O, then air-dried. Slides were coverslipped with DPX neutral mounting medium (Thermo Fisher Scientific). Cells were imaged on a Nikon 90i microscope fitted with a DXM1200C camera, at 1000x magnification.

***In vitro* developmental potential (colony forming) assays**:

GMPs, EoPs and CD11b+Siglec-F+IL5Rα- cells were sorted from the bone marrow (following red cell lysis) of C57BL/6 mice, using the stains and surface markers as described above. Colony assays were performed in triplicate as described by [5]. Briefly, 200 or 2000 cells were mixed with 1x MOD DME, 20% FCS 289, 0.3% Bacto Agar, murine stem cell factor (100 ng/mL, WEHI), murine interleukin 3 (10 ng/mL, WEHI) and human erythropoietin (2 U/mL, Eprex, Janssen Pharmaceuticals) and plated in 35 mm petri dishes. Dishes were incubated for 7 d in humidified air at 37°C, 10% CO_2. Agar cultures were then fixed with 2.5% glutaraldehyde, floated onto glass slides and air-dried. Slides were stained for acetylcholinesterases, and with Luxol Fast Blue and hematoxylin as previously described [5], and scored by viewing on a Nikon Optiphot-2 light microscope.

***In vivo* developmental potential assays**:

Total bone marrow was flushed from the hips, femurs and tibiae of three 14 week old UBC-GFP mice in PBS/2% FCS, triturated using a 23 gauge needle, and overlaid onto 60% Percoll in PBS. Cells were centrifuged at room temperature at 400 g for 25 min. Cells at the Percoll interface were collected, washed twice with PBS/2% FCS, stained at 10^8 cells/mL with a cocktail of fluorescently-conjugated antibodies against IL5Rα, Siglec-F, CD11b, CD34, unlabeled CD16/32. Prior to sorting, cells were resuspended in PBS supplemented with 2% FCS, 2 mM EDTA, 1 μg/mL propidium iodide (Sigma). The CD11b+Siglec-F+IL5Rα- population was isolated by cell sorting on a BD FACS Aria III (BD Biosciences). Sorted cells were washed once with ice-cold PBS and resuspended in a 25 μL volume of ice-cold PBS.

Recipient Ly5.1 mice were sub-lethally irradiated (550 rad) 24 h prior to transplantation. Analgesia (Temgesic, 100μg/kg, i.p.) was administered to recipient mice 30 min prior to surgery. At the time of surgery, mice were anaesthetized with isoflurane via anaesthetic machine, the skin next to surgery site shaved and a 1 cm incision through skin and peritoneum made. Sorted cells (25 μL) were injected under the capsule of the sinus of the spleen, and the site pressed with a sterile cotton bud to prevent leakage of the sample and mild bleeding. A mock recipient mouse was injected with 25 μL PBS alone. A drop of Histoacryl (B. Braun) was then used to seal the injection site and the spleen placed back into the peritoneum. The peritoneum and skin were separately sutured, and wound clips applied to outer skin. Further analgesia (Temgesic, 100μg/kg, i.p.) was administered 90 min after surgery. Spleens were collected from recipient mice 1 and 3 d after surgery and prepared for flow cytometry as described above.

RNA-sequencing:

Siglec-F+IL5Rα- cells, CMPs, GMPs EoPs and Eosinophils were sorted from the BM of 6-10 week old C57BL/6 and 6 week old $Myb^{Plt4/Plt4}$ mice as described above, resuspended and stored in 75-350 μL RLT buffer as per manufacturer's instructions (Qiagen). Total RNA was isolated using the RNAeasy Micro Kit (Qiagen) according to manufacturer's instructions. RNA quality was assessed using an Agilent 2100 Bioanalyzer. 130-200 ng total RNA per sample was submitted to the Australian Genome Research Facility (AGRF) for high throughput mRNA-sequencing (RNA-seq). Messenger RNA libraries were synthesized using Illumina's TruSeq Stranded mRNA sample preparation protocol, and 100 bp single or paired end reads generated

by high-throughput sequencing with an Illumina HiSeq 2500 (Illumina). 2-5 independent RNA samples (biological replicates) per cell type were sequenced.
Reads were aligned to the *Mus musculus* genome (Ens84, GRCm38) using the Rsubread package [6] and assigned to genes by the featureCounts function [7] using the Ensembl annotation. Filtering and normalization used the edgeR package [8]. Genes with a count per million (CPM) of at least 1 in 2 or more samples were retained for further analysis. Compositional differences between libraries were normalized using the trimmed mean of *M*-values (TMM) method [9]. Subsequent differential expression analysis was performed using the limma package [10]. Counts were transformed to log2-CPM values (with an offset of 0.5) with associated observational and sample-specific weights obtained from the voomWithQualityWeights method [11] assuming a linear model [12] with effects for cell type. p-values were corrected for multiple testing using the method of Benjamini and Hochberg [13].
Contrasts between the different cell-types were estimated and differential expression was tested relative to a fold-change of 1.5 using TREAT [14] and a false discovery rate (FDR) cut-off of 0.05. Heatmaps of $\log_2$ counts per million (CPM) were generated for various sets of genes (100 most variable across all samples, 100 most differentially expressed genes based on FDR) using the heatmap.2 function from the gplots R package. Multidimensional scaling of the counts using the 500 most variable genes between each pair of samples was used to explore the relationships between samples.
This dataset has been submitted to the Gene Expression Omnibus under the accession number GSE107495.

Gene ontology analyses:

Gene ontology analyses were performed on lists of differentially expressed genes using the MSigDB tab of the Broad Institute's online GSEA software, selecting for analyses on C5 GO_Gene_Sets [15, 16]. The curated list of transcription factors (TFs) published in [17] were used in the identification of differentially expressed transcription factors.

References

1. Schaefer, B. C., Schaefer, M. L., Kappler, J. W., Marrack, P., Kedl, R. M. (2001) Observation of antigen-dependent CD8+ T-cell/ dendritic cell interactions in vivo. Cellular immunology 214, 110-22.
2. Carpinelli, M. R., Hilton, D. J., Metcalf, D., Antonchuk, J. L., Hyland, C. D., Mifsud, S. L., Di Rago, L., Hilton, A. A., Willson, T. A., Roberts, A. W., Ramsay, R. G., Nicola, N. A., Alexander, W. S. (2004) Suppressor screen in Mpl-/- mice: c-Myb mutation causes supraphysiological production of platelets in the absence of thrombopoietin signaling. Proceedings of the National Academy of Sciences of the United States of America 101, 6553-8.
3. Lee, J. J., Dimina, D., Macias, M. P., Ochkur, S. I., McGarry, M. P., O'Neill, K. R., Protheroe, C., Pero, R., Nguyen, T., Cormier, S. A., Lenkiewicz, E., Colbert, D., Rinaldi, L., Ackerman, S. J., Irvin, C. G., Lee, N. A. (2004) Defining a link with asthma in mice congenitally deficient in eosinophils. Science 305, 1773-6.
4. Dent, L. A., Strath, M., Mellor, A. L., Sanderson, C. J. (1990) Eosinophilia in transgenic mice expressing interleukin 5. The Journal of experimental medicine 172, 1425-31.
5. Alexander, W. S., Roberts, A. W., Nicola, N. A., Li, R., Metcalf, D. (1996) Deficiencies in progenitor cells of multiple hematopoietic lineages and defective megakaryocytopoiesis in mice lacking the thrombopoietic receptor c-Mpl. Blood 87, 2162-70.
6. Liao, Y., Smyth, G. K., Shi, W. (2013) The Subread aligner: fast, accurate and scalable read mapping by seed-and-vote. Nucleic acids research 41, e108.
7. Liao, Y., Smyth, G. K., Shi, W. (2014) featureCounts: an efficient general purpose program for assigning sequence reads to genomic features. Bioinformatics 30, 923-30.
8. Robinson, M. D., McCarthy, D. J., Smyth, G. K. (2010) edgeR: a Bioconductor package for differential expression analysis of digital gene expression data. Bioinformatics 26, 139-40.
9. Robinson, M. D. and Oshlack, A. (2010) A scaling normalization method for differential expression analysis of RNA-seq data. Genome biology 11, R25.

10. Ritchie, M. E., Phipson, B., Wu, D., Hu, Y., Law, C. W., Shi, W., Smyth, G. K. (2015) limma powers differential expression analyses for RNA-sequencing and microarray studies. Nucleic acids research 43, e47.
11. Liu, R., Holik, A. Z., Su, S., Jansz, N., Chen, K., Leong, H. S., Blewitt, M. E., Asselin-Labat, M. L., Smyth, G. K., Ritchie, M. E. (2015) Why weight? Modelling sample and observational level variability improves power in RNA-seq analyses. Nucleic acids research 43, e97.
12. Smyth, G. K. (2004) Linear models and empirical bayes methods for assessing differential expression in microarray experiments. Statistical applications in genetics and molecular biology 3, Article3.
13. Benjamini, Y. and Hochberg, Y. (1995) Controlling the False Discovery Rate - a Practical and Powerful Approach to Multiple Testing. J Roy Stat Soc B Met 57, 289-300.
14. McCarthy, D. J. and Smyth, G. K. (2009) Testing significance relative to a fold-change threshold is a TREAT. Bioinformatics 25, 765-71.
15. Mootha, V. K., Lindgren, C. M., Eriksson, K. F., Subramanian, A., Sihag, S., Lehar, J., Puigserver, P., Carlsson, E., Ridderstrale, M., Laurila, E., Houstis, N., Daly, M. J., Patterson, N., Mesirov, J. P., Golub, T. R., Tamayo, P., Spiegelman, B., Lander, E. S., Hirschhorn, J. N., Altshuler, D., Groop, L. C. (2003) PGC-1alpha-responsive genes involved in oxidative phosphorylation are coordinately downregulated in human diabetes. Nature genetics 34, 267-73.
16. Subramanian, A., Tamayo, P., Mootha, V. K., Mukherjee, S., Ebert, B. L., Gillette, M. A., Paulovich, A., Pomeroy, S. L., Golub, T. R., Lander, E. S., Mesirov, J. P. (2005) Gene set enrichment analysis: a knowledge-based approach for interpreting genome-wide expression profiles. Proceedings of the National Academy of Sciences of the United States of America 102, 15545-50.
17. Zhang, J. A., Mortazavi, A., Williams, B. A., Wold, B. J., Rothenberg, E. V. (2012) Dynamic transformations of genome-wide epigenetic marking and transcriptional control establish T cell identity. Cell 149, 467-82.

Supplementary Figure 1

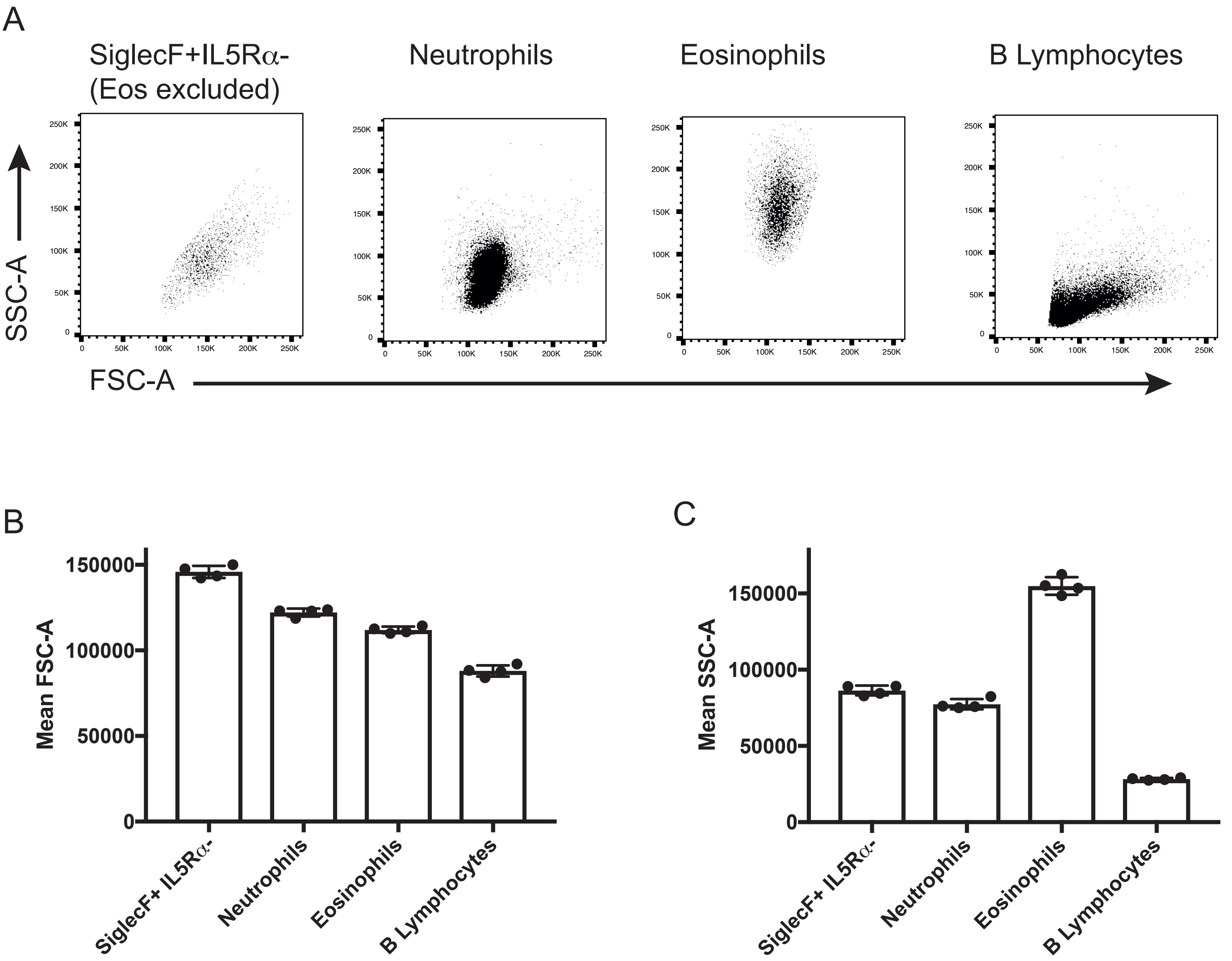

Supplementary Figure 1: Quantification of scatter in eosinophils (CD11b+SiglecF+IL5RαInt, SSChi), neutrophils (CD11b+SiglecF-Ly6G+), B lymphocytes (B220+) and CD11b+ SiglecF+ IL5Rα- cells (A) Representative dot plots showing forward scatter (FSC) and side scatter (SSC) of cell populations. (B) Quantification of FSC (C) Quantification of SSC. Data are presented as Mean +/- SEM (N=4). Data points for individual mice are shown.
